# A probabilistic approach to visualize the effect of missing data on PCA in ancient human genomics

**DOI:** 10.1186/s12864-025-11728-1

**Published:** 2025-05-27

**Authors:** Susanne Zabel, Samira Breitling, Cosimo Posth, Kay Nieselt

**Affiliations:** 1 Institute for Bioinformatics and Medical Informatics, University of Tübingen, Sand 14, 72076 Tübingen, Germany; 2 Archaeo- and Palaeogenetics, Institute for Archaeological Sciences, Department of Geosciences, University of Tübingen, Hölderlinstraße 12, 72074 Tübingen, Germany; 3 Senckenberg Centre for Human Evolution and Palaeoenvironment, University of Tübingen, Hölderlinstraße 12, 72074 Tübingen, Germany

**Keywords:** Ancient genomics, Missing data, Population genetics, Principal component analysis, SmartPCA, Uncertainty

## Abstract

**Background:**

Principal Component Analysis (PCA) is widely used in population genetics to visualize genetic relationships and population structures. In ancient genomics, genotype information may in parts remain unresolved due to the low abundance and degraded quality of ancient DNA. While methods like SmartPCA allow the projection of ancient samples despite missing data, they do not quantify projection uncertainty. The reliability of PCA projections for often very sparse ancient genotype samples is not well understood. Ignoring this uncertainty may lead to overconfident conclusions about the observed genetic relationships and population structure.

**Results:**

This study systematically investigates the impact of missing loci on PCA projections using both simulated and real ancient human genotype data. Through extensive simulations with high-coverage ancient samples, we demonstrate that increasing levels of missing data can lead to less accurate SmartPCA projections, highlighting the importance of considering uncertainty when interpreting PCA results from ancient samples. To address this, we developed a probabilistic framework to quantify the uncertainty in PCA projections due to missing data. By applying our methodology to modern and ancient West Eurasian genotype samples from the Allen Ancient DNA Resource database, we could show a high concordance between our predicted projection and empirically derived distributions. Applying this framework to real-world data, we demonstrate its utility in predicting and visualizing embedding uncertainties for ancient samples of varying SNP coverages.

**Conclusion:**

Our results emphasize the importance of accounting for projection uncertainty in ancient population studies. We therefore make our probabilistic model available through TrustPCA, a user-friendly web tool that provides researchers with uncertainty estimates alongside PCA projections, facilitating data exploration in ancient human genomic studies and enhancing transparency in data quality reporting.

**Supplementary Information:**

The online version contains supplementary material available at 10.1186/s12864-025-11728-1.

## Background

Human curiosity about our origins has driven research into fundamental questions about where we come from and how we are related to each other. Population genetics, the study of genetic composition and variation within and between populations, aims to understand how genetic diversity evolves over time. The analysis of ancient DNA has amplified this effort, offering insights into past genetic diversity and human evolution. A central challenge in this field is to interpret the ancestral relationships between individuals and populations sampled, especially when working with high-dimensional genotype data. To address this challenge, dimensionality reduction techniques and the resulting visualizations have become a central part of analyses in population genetics [1].

Among these techniques, principal component analysis (PCA) [2, 3] stands out as the most widely used method for reducing the dimensionality of genotype data. PCA works by projecting samples onto a subspace defined by principal components (PCs), which capture the directions of maximum variance in the data. The coordinates of the samples in the reduced space, computed as linear combinations of their original allelic or genotypic values, offer clear genetic interpretations [4, 5]. Typically, only the first two or three PCs are visualized in scatter plots, as they capture most of the variance and effectively reveal patterns of population structure, ancestry, and relatedness among individuals [4, 5].

A significant challenge in analyzing ancient genotype data is the low abundance and degraded quality of ancient DNA [6], which results in sparse data that make direct application of PCA impractical [7]. To overcome this, PCA is often performed on complete modern datasets to define the principal axes, onto which ancient samples are projected using algorithms capable of handling missing data [8]. The most popular PCA software in population genetics that implements this approach is SmartPCA [9], which is part of the EIGENSOFT software suite [9, 10].

However, the reliability, robustness, and replicability of PCA in population genetic studies have been called into question in recent years due to

- the potential for unreliable and misleading projections that do not accurately reflect genetic distances [11],
- the inability to infer admixture levels or directions from PCA plots [11, 12],
- the heavy dependence of results on the choice of reference populations [4, 11, 13, 14],

For studies involving ancient samples, these issues are exacerbated, as the interpretation of admixture and the origins of populations often hinges on the selection of modern reference datasets [11]. Despite these challenges, PCA remains the most widely used method for dimensionality reduction in population genetics.

Modern and ancient genomes are often genotyped at ${\sim }$600,000 autosomal single nucleotide polymorphisms (SNPs) using the Human Origins array [15]. However, for ancient samples, the proportion of observed positions can be lower than 1 % of the array [16]. The reliability of PCA projections for such highly sparse samples is not well understood, leaving their placement on PCA plots to the experience of researchers or subjective judgment.

In this work, we systematically analyze how varying levels of missing SNPs influence the reliability of SmartPCA projections through simulations. These simulations aim to raise awareness of how far SmartPCA estimates can deviate from the true embedding. Building on this, we introduce a probabilistic model to quantify embedding uncertainty, offering users of SmartPCA insights into the reliability of projections based on the observed genotypes of ancient individuals. While the embedding generated by SmartPCA represents the best estimate given the limited number of observed SNPs [8], our model provides a probability distribution around this estimate. This distribution indicates the likelihood of the sample being projected to a different place on the map if all SNPs were known, offering a more comprehensive understanding of the uncertainty in PCA results. Since SmartPCA differs methodologically from conventional PCA, existing uncertainty-aware PCA methods (e.g., [17, 18]) cannot be directly applied in this setting. Our approach uniquely addresses this gap by specifically modeling projection uncertainty in SmartPCA. To facilitate accessibility, we developed TrustPCA, a user-friendly tool that implements our probabilistic model, allowing researchers to evaluate and visualize projection uncertainty in their analyses.

## Material & methods

### Data, code, and software availability

The data that support the findings of this study are openly available from the Allen Ancient DNA Resource (AADR), version v54.1.p1: https://doi.org/10.7910/DVN/FFIDCW) [16]. The source code for the proposed methodology and for the analyses described in this paper are available at https://github.com/Integrative-Transcriptomics/TrustPCA/tree/theory_and_paper_code. TrustPCA is openly available at https://trustpca-tuevis.cs.uni-tuebingen.de/, with the source code accessible at https://github.com/Integrative-Transcriptomics/TrustPCA/tree/main.

### Data

In this study, we utilized genotype data from the *Allen Ancient DNA Resource* (AADR) [16], Version v54.1.p1, released on March 6, 2023. Specifically, we used the *1240 K+HO* dataset, which includes genotype data for 20,503 unique human individuals (9,990 ancient and 10,513 present-day) based on the Human Origins array [15], covering 597,573 sites. The genotype data is provided in EIGENSTRAT format [9, 10], consisting of a tabular genotype file where columns represent individuals and rows represent genotype sites. The table entries can have one of four numbers (0, 1, 2, or 9), representing zero (0), one (1), or two (2) copies of the reference allele. Missing genotypes are encoded as 9 [9, 10].

From the AADR database, all individuals originating from 67 commonly used modern West Eurasian populations (see Additional file 1 & 2, [19]) were selected, totaling 1,433 individuals. These individuals were used to infer genetic variation. Additionally, all ancient West Eurasian individuals from the Mesolithic or later periods were included in the analysis, resulting in a dataset of 6,627 ancient individuals (see Additional file 4).

It is important to note that genotype calls for all ancient individuals are pseudo-haploid, whereas those for modern individuals are diploid. Additionally, SNP coverage—defined as the proportion of successfully genotyped single nucleotide polymorphisms (SNPs) per individual relative to the total of 540,247 variant SNPs analyzed in this study (see section “Computation of the PC space”)—is uniformly high for modern individuals (100 %), but varies widely among ancient individuals, ranging from 1 % to 100 %. The full distribution of SNP coverage across all ancient individuals is shown in Figure S1.

### Principal component analysis

Principal Component Analysis (PCA) [2, 3] is a dimensionality reduction method that can provide a compact representation of data with minimal loss of information. Variables in a high dimensional data matrix may be correlated, and closely fit a linear manifold in a lower dimension. Objects in a dataset often depend only on these few linear, uncorrelated variables. PCA reveals these representations in terms of principal components, which capture in decreasing order the most variation in the data.

Consider a data matrix ${\varvec{X}} \in \mathbb {R}^{D \times N}$ where rows represent features and columns represent observations, so that each of the *N* observations is defined by a *D*-dimensional feature vector $\{{\varvec{x}}_i\}_{i=1,\ldots , N}$, with ${\varvec{x}}_i \in \mathbb {R}^D$. We assume that the data is centered, with each feature having a mean of zero. PCA performs dimensionality reduction by projecting the original *D*-dimensional feature vectors onto a *P*-dimensional subspace ($P \le D$). This subspace is spanned by *P* orthonormal vectors, known as the principal components (PCs), and denoted as $\{{\varvec{v}}_k\}_{k=1,\ldots ,P}$, with ${\varvec{v}}_k \in \mathbb {R}^D$. The projections $\{{\varvec{t}}_i\}_{i=1,\ldots ,N}$, where each ${\varvec{t}}_i \in \mathbb {R}^P$, are obtained via an orthogonal linear transformation. Specifically, the projection of a data point ${\varvec{x}}_i$ onto the *k*-th direction is given by:

1 $$\begin{aligned} {\varvec{t}}_{ki} = {\varvec{v}}_k^\intercal {\varvec{x}}_i \end{aligned}$$

In matrix form, this transformation can be expressed as:

2 $$\begin{aligned} {\varvec{t}}_i = {\varvec{V}}^\intercal {\varvec{x}}_i \end{aligned}$$

where ${\varvec{V}} \in \mathbb {R}^{D \times P}$ is the matrix whose columns are the orthonormal vectors ${\varvec{v}}_k$. The PCs are selected to maximize the sum of variances of the projections. It can be shown that the optimal PCs correspond to the *P* eigenvectors $v_k$ of the covariance matrix ${\varvec{C}} = \frac{1}{n-1}{\varvec{X}}^{\intercal }{\varvec{X}}$, associated with the *P* largest eigenvalues [2].

For large datasets, the calculation of the covariance matrix can be computationally expensive. A faster and numerically more stable method is to factorize ${\varvec{X}}$ by a singular value decomposition (SVD),

3 $$\begin{aligned} {\varvec{X}} = {\varvec{U}} {\varvec{\Sigma }} {\varvec{V}}^\intercal , \end{aligned}$$

where ${\varvec{U}} \in \mathbb {R}^{N \times N}$, ${\varvec{\Sigma }} \in \mathbb {R}^{N \times D}$, and ${\varvec{V}} \in \mathbb {R}^{D \times D}$. Note that the columns of ${\varvec{V}}$ are the eigenvectors of ${\varvec{X}}^\intercal {\varvec{X}}$. The diagonal entries $\sigma _i = {\varvec{\Sigma }}_{ii}$ of ${\varvec{\Sigma }}$ are known as the singular values of ${\varvec{X}}$. These singular values indicate the importance of each principal component in explaining data variance. The rank *Q* of ${\varvec{X}}$ determines the number of non-zero singular values. Typically, the first $P \le Q$ eigenvectors are selected to form the reduced representation, as they capture the majority of the variance in the data. In many cases, only the first two eigenvectors ($P=2$) are considered.

### SmartPCA: applying PCA to genotype data

Several software tools, including EIGENSOFT [9, 10] and PLINK [20], have been developed to implement PCA specifically for genetic data. Among these, we focus on SmartPCA [9], a module within the EIGENSOFT suite that efficiently performs PCA on genotype data and has become the standard tool in population genetic studies.

In this context, the features of the data matrix ${\varvec{M}}$ represent genotype information across various loci. Prior to PCA application, the data is centered and optionally scaled. In this work, data was scaled to account for the genetic drift, reflecting the rate of allele frequency change at each locus [9]:

4 $$\begin{aligned} {\varvec{X}}_{ij} = \frac{{\varvec{M}}_{ij} - {\varvec{\mu }}_j}{\sqrt{{\varvec{p}}_j (1-{\varvec{p}}_j)}} \quad \text {with} \quad {\varvec{p}}_j = \frac{{\varvec{\mu }}_j}{2}, \end{aligned}$$

where ${\varvec{\mu }}_j$ is the mean of feature *j*. Please note that the “shrinkmode: YES” option of the SmartPCA program was not selected.

Following this normalization, PCA is performed on the centered and scaled data matrix ${\varvec{M}}$ to compute principal components and to project the samples into a low-dimensional space.

This PCA methodology is predominantly applied to high-quality genotype data obtained from modern populations, where complete genotypic information is generally available across all loci of interest. For ancient samples, which often lack full genotype resolution, direct PCA application is challenging. Instead, a common approach is to project ancient samples onto the top principal components (typically the first two) derived from modern populations, thus enabling comparative analyses without requiring complete genotypic data. This method, known as *projection to the model plane* [8], is implemented in SmartPCA and is described below.

Given the eigenvector matrix ${\varvec{V}} \in \mathbb {R}^{D \times Q}$ derived from a modern population, an ancient genotype sample ${\varvec{z}} \in \mathbb {R}^D$ can be divided into observed genotypes ${\varvec{z}}^* \in \mathbb {R}^{M}$, where $0> M\le D$, and unobserved genotypes ${\varvec{z}}^{\#} \in \mathbb {R}^{D-M}$. To project ${\varvec{z}}$ onto the principal component space defined by ${\varvec{V}}$, the following procedure is applied: First, ${\varvec{z}}^*$ is centered and scaled using the mean and genetic drift calculated from the modern population (Eq. 4). With the centered and scaled ${\varvec{z}}^*$, the projection scores $\hat{{\varvec{\tau }}} \in \mathbb {R}^P$ are computed, representing the coordinates of the sample in the principal component space. Since ${\varvec{z}}$ contains missing data, $\hat{{\varvec{\tau }}}$ is obtained as a least-square estimate [8]:

5 $$\begin{aligned} \hat{{\varvec{\tau }}} = ({\varvec{V}}^{*\intercal } {\varvec{V}}^*)^{-1} {\varvec{V}}^{*\intercal } {\varvec{z}}^*, \end{aligned}$$

where ${\varvec{V}}^* \in \mathbb {R}^{M\times Q}$ is the subset of rows of ${\varvec{V}}$ corresponding to the observed loci in ${\varvec{z}}$ (thus ${\varvec{z}}^*$). The result, $\hat{{\varvec{\tau }}}$, provides an approximation of the true embedding ${\varvec{\tau }}$ that would have been obtained if all genotypes were observed. The factor $({\varvec{V}}^{*\intercal }{\varvec{V}}^*)^{-1}$ minimizes the sum of the quadratic differences between the observed values and the predicted model values. This ensures an effective regression of the observed parts of the sample onto the PCA model plane and takes the entire structure into account.

### Evaluating the impact of missing data

**Fig. 1:**
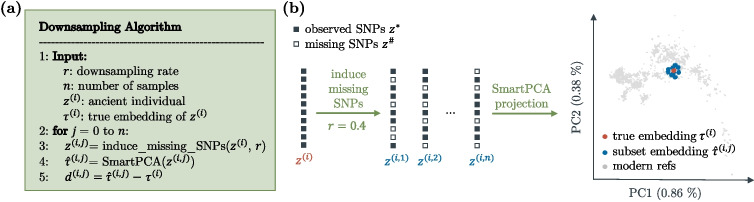
Simulation of missing genotypes. **a** Algorithm to simulate missing genotypes in ancient individuals. Starting with a high-coverage sample ${\varvec{z}}^{(i)}$ and its SmartPCA embedding ${\varvec{\tau }}^{(i)}$, considered the true embedding of ${\varvec{z}}^{(i)}$, the algorithm generates *n* realizations ${\varvec{z}}^{(i,j)}$ by randomly omitting genotypes at a specified rate *r*. Each realization ${\varvec{z}}^{(i,j)}$ is projected onto a predefined PCA subspace, yielding *n* estimators $\hat{{\varvec{\tau }}}^{(i,j)}$ for ${\varvec{\tau }}^{(i)}$. The distribution of $\hat{{\varvec{\tau }}}^{(i,j)}$ around ${\varvec{\tau }}^{(i)}$ is analyzed both visually and statistically. **b** Schematic representation of the downsampling algorithm, illustrating the process of random genotype omission and SmartPCA projection

To assess the effect of missing genotype information in ancient samples on the projection using the SmartPCA approach as described above, a comprehensive simulation experiment was performed. Fifteen high-coverage ancient samples ${\varvec{z}}^{(i)}$, $i=1,\ldots ,15$, each with less than 10 % missing genotypes, were selected. These samples were harmonized—aligned to a common set of SNPs (union)—to ensure consistency in the proportion of missing loci. Random omissions were then introduced to simulate varying levels of missing data across the samples. Each ancient sample was projected onto a two-dimensional subspace derived from modern West Eurasian samples using SmartPCA, yielding embeddings ${\varvec{\tau }}^{(i)}$, which were considered accurate representations of the samples’ true positions in the subspace (Fig. 1a). To simulate the impact of missing data, genotypes were randomly omitted from each ancient sample at a specified rate *r*. This process was repeated *n* times, generating multiple realizations $\{{\varvec{z}}^{(i, j)}\}_{j=1,\ldots ,n}$ of each sample with varying patterns of missing data (Fig. 1b). The modified samples ${\varvec{z}}^{(i, j)}$ were then projected using SmartPCA, resulting in a set of embeddings, $\{\hat{{\varvec{\tau }}}^{(i, j)}\}_{j=1,\ldots ,n}$. Each embedding $\hat{{\varvec{\tau }}}^{(i, j)}$ serves as an approximation of the original embedding ${\varvec{\tau }}^{(i)}$. To quantify the effect of missing data on the projection, statistics about the difference $\hat{{\varvec{\tau }}}^{(i, j)} - {\varvec{\tau }}^{(i)}$ were computed and analyzed for each principal component of the projection.

### Modeling the deviation between estimated and true embeddings in PCA

This section introduces a method to estimate a probability distribution over the true position—if all genotypes were observed—of a sample with unobserved genotypes in the principal component (PC) space. This distribution quantifies the potential deviation of the estimated embedding obtained through SmartPCA from the true embedding, capturing the uncertainty introduced by missing genotypes. The degree of uncertainty depends on the amount of missing data and the strength of the signals provided by the observed loci.

The original publication of the ‘*projection to the model plane*’ method [8] provides an error analysis for the PC score estimation arising from missing data, serving as the foundation for the proposed methodology to estimate embedding uncertainty.

In the following, let ${\mathcal {P}} = \{1, \ldots , P\}$ be the set of indices of the principal components of interest, and let ${\mathcal {Q}} = \{P+1, \ldots , Q\}$ be the set of indices of the remaining components. For instance, ${\varvec{V}}_{\mathcal {P}} \in \mathbb {R}^{D\times P}$ is a matrix containing the first *P* eigenvectors, and ${\varvec{\tau }}_{{\mathcal {P}}}\in \mathbb {R}^P$ represents the principal component scores of a sample in this subspace. The discrepancy between the true embedding ${\varvec{\tau }}_{{\mathcal {P}}}$ and its estimate by SmartPCA $\hat{{\varvec{\tau }}}_{{\mathcal {P}}}$ is given by [8]

6 $$\begin{aligned} {\varvec{\tau }}_{{\mathcal {P}}} - \hat{{\varvec{\tau }}}_{{\mathcal {P}}} & = {\varvec{\tau }}_{{\mathcal {P}}} - ({\varvec{V}}_{{\mathcal {P}}}^{* \intercal }{\varvec{V}}_{{\mathcal {P}}}^{*})^{-1} {\varvec{V}}_{{\mathcal {P}}}^{* \intercal } {\varvec{z}}^{*} \end{aligned}$$

7 $$\begin{aligned} & = {\varvec{\tau }}_{{\mathcal {P}}} - ({\varvec{V}}_{{\mathcal {P}}}^{* \intercal }{\varvec{V}}_{{\mathcal {P}}}^{*})^{-1} {\varvec{V}}_{{\mathcal {P}}}^{* \intercal } {\varvec{V}}^{*} {\varvec{\tau }}\end{aligned}$$

8 $$\begin{aligned} & = {\varvec{\tau }}_{{\mathcal {P}}} - [({\varvec{V}}_{{\mathcal {P}}}^{* \intercal }{\varvec{V}}_{{\mathcal {P}}}^{*})^{-1} {\varvec{V}}_{{\mathcal {P}}}^{* \intercal } ({\varvec{V}}_{{\mathcal {P}}}^{*} {\varvec{\tau }}_{{\mathcal {P}}} + {\varvec{V}}_{{\mathcal {Q}}}^{*} {\varvec{\tau }}_{{\mathcal {Q}}})]\end{aligned}$$

9 $$\begin{aligned} & = {\varvec{\tau }}_{{\mathcal {P}}} - ({\varvec{V}}_{{\mathcal {P}}}^{* \intercal }{\varvec{V}}_{{\mathcal {P}}}^{*})^{-1} {\varvec{V}}_{{\mathcal {P}}}^{* \intercal } {\varvec{V}}_{{\mathcal {P}}}^{*} {\varvec{\tau }}_{{\mathcal {P}}} - ({\varvec{V}}_{{\mathcal {P}}}^{* \intercal }{\varvec{V}}_{{\mathcal {P}}}^{*})^{-1} {\varvec{V}}_{{\mathcal {P}}}^{* \intercal } {\varvec{V}}_{{\mathcal {Q}}}^{*} {\varvec{\tau }}_{{\mathcal {Q}}}\end{aligned}$$

10 $$\begin{aligned} & = - ({\varvec{V}}_{\mathcal {P}}^{* \intercal }{\varvec{V}}_{\mathcal {P}}^{*})^{-1} {\varvec{V}}_{\mathcal {P}}^{* \intercal } {\varvec{V}}_{\mathcal {Q}}^{*} {\varvec{\tau }}_{\mathcal {Q}}. \end{aligned}$$

Note that ${\varvec{z}}^*={\varvec{V}}^*{\varvec{\tau }}$ follows directly from rearranging ${\varvec{\tau }} = {\varvec{V}}^{* \intercal } {\varvec{z}}$ (Eq. 2), leveraging the fact that ${\varvec{V}}^*$ is an orthogonal matrix (Eqs. 6 to 7). Additionally, observe that $({\varvec{V}}_{{\mathcal {P}}}^{* \intercal }{\varvec{V}}_{{\mathcal {P}}}^{*})^{-1} {\varvec{V}}_{{\mathcal {P}}}^{* \intercal } {\varvec{V}}_{{\mathcal {P}}}^{*}$ reduces to the identity matrix since ${\varvec{V}}_{{\mathcal {P}}}^{* \intercal }{\varvec{V}}_{{\mathcal {P}}}^{*}$ is an invertible square matrix (Eqs. 9 to 10).

The derived expression for the discrepancy, ${\varvec{\tau }}_{{\mathcal {P}}} - \hat{{\varvec{\tau }}}_{{\mathcal {P}}}$, shows a linear dependence on ${\varvec{\tau }}_{\mathcal {Q}}$. Therefore, we can use the simple rules of moment propagation that can be applied in linear settings to obtain an analytical exact solution [21]. The expectation of the PC scores $\mathbb {E}[{\varvec{\tau }}_{\mathcal {Q}}]$ is zero, i.e., $\mathbb {E}[{\varvec{\tau }}_{\mathcal {Q}}] = \varvec{0}$, as a consequence of centering the data feature-wise prior to performing PCA. Propagating this expectation through the linear function yields the expectation of the discrepancy:

11 $$\begin{aligned} \mathbb {E}[{\varvec{\tau }}_{{\mathcal {P}}} - \hat{{\varvec{\tau }}}_{{\mathcal {P}}}] & = - ({\varvec{V}}_{\mathcal {P}}^{* \intercal }{\varvec{V}}_{\mathcal {P}}^{*})^{-1} {\varvec{V}}_{\mathcal {P}}^{* \intercal } {\varvec{V}}_{\mathcal {Q}}^{*} \mathbb {E}[{\varvec{\tau }}_{\mathcal {Q}}] \end{aligned}$$

12 $$\begin{aligned} & = - ({\varvec{V}}_{\mathcal {P}}^{* \intercal }{\varvec{V}}_{\mathcal {P}}^{*})^{-1} {\varvec{V}}_{\mathcal {P}}^{* \intercal } {\varvec{V}}_{\mathcal {Q}}^{*} \varvec{0}\end{aligned}$$

13 $$\begin{aligned} & = \varvec{0}. \end{aligned}$$

The variances of the PC scores, $\mathbb {V}[{\varvec{\tau }}_{\mathcal {Q}}] \in \mathbb {R}^{Q \times Q}$, are determined by the eigenvalues corresponding to the components, which are equal to the variances of the principal components. Because the PCs are uncorrelated, all covariances between components are zero. Therefore, the covariance matrix $\mathbb {V}[{\varvec{\tau }}_{\mathcal {Q}}]$ is diagonal and can be expressed as ${\varvec{\Lambda }}_{\mathcal {Q}}$, where ${\varvec{\Lambda }}_{\mathcal {Q}}$ contains the eigenvalues of the *Q* corresponding components. Propagating the variance through the linear function yields the variance of the discrepancy:

14 $$\begin{aligned} \mathbb {V}[{\varvec{\tau }}_{{\mathcal {P}}} - \hat{{\varvec{\tau }}}_{{\mathcal {P}}}] & = - ({\varvec{V}}_{\mathcal {P}}^{* \intercal }{\varvec{V}}_{\mathcal {P}}^{*})^{-1} {\varvec{V}}_{\mathcal {P}}^{* \intercal } {\varvec{V}}_{\mathcal {Q}}^{*} \mathbb {V}[{\varvec{\tau }}_{\mathcal {Q}}] (- ({\varvec{V}}_{\mathcal {P}}^{* \intercal }{\varvec{V}}_{\mathcal {P}}^{*})^{-1} {\varvec{V}}_{\mathcal {P}}^{* \intercal } {\varvec{V}}_{\mathcal {Q}}^{*})^\intercal \end{aligned}$$

15 $$\begin{aligned} & = - ({\varvec{V}}_{\mathcal {P}}^{* \intercal }{\varvec{V}}_{\mathcal {P}}^{*})^{-1} {\varvec{V}}_{\mathcal {P}}^{* \intercal } {\varvec{V}}_{\mathcal {Q}}^{*} {\varvec{\Lambda }}_{\mathcal {Q}} (- ({\varvec{V}}_{\mathcal {P}}^{* \intercal }{\varvec{V}}_{\mathcal {P}}^{*})^{-1} {\varvec{V}}_{\mathcal {P}}^{* \intercal } {\varvec{V}}_{\mathcal {Q}}^{*})^\intercal . \end{aligned}$$

Assuming the discrepancy is Gaussian distributed, it is fully specified by its expectation and variance. From the expectation and the variance of the discrepancy ${\varvec{\tau }}_{{\mathcal {P}}} - \hat{{\varvec{\tau }}}_{{\mathcal {P}}}$, we can derive those of the true embedding ${\varvec{\tau }}_{{\mathcal {P}}}$. Since the expectation of the discrepancy, $\mathbb {E}[{\varvec{\tau }}_{{\mathcal {P}}} - \hat{{\varvec{\tau }}}_{{\mathcal {P}}}]$, is zero, the expectation for the true embedding ${\varvec{\tau }}_{{\mathcal {P}}}$ can be expressed as:

16 $$\begin{aligned} \mathbb {E}[{\varvec{\tau }}_{{\mathcal {P}}} - \hat{{\varvec{\tau }}}_{{\mathcal {P}}}] & = \varvec{0}\end{aligned}$$

17 $$\begin{aligned} \mathbb {E}[{\varvec{\tau }}_{{\mathcal {P}}}] - \mathbb {E}[\hat{{\varvec{\tau }}}_{{\mathcal {P}}}] & = \varvec{0}\end{aligned}$$

18 $$\begin{aligned} \mathbb {E}[{\varvec{\tau }}_{{\mathcal {P}}}] - \hat{{\varvec{\tau }}}_{{\mathcal {P}}} & = \varvec{0}\end{aligned}$$

19 $$\begin{aligned} \mathbb {E}[{\varvec{\tau }}_{{\mathcal {P}}}] & = \hat{{\varvec{\tau }}}_{{\mathcal {P}}} \end{aligned}$$

Since $\hat{{\varvec{\tau }}}_{{\mathcal {P}}}$ is a known (observed) point estimate, we have $\mathbb {E}[\hat{{\varvec{\tau }}}_{{\mathcal {P}}}] = \hat{{\varvec{\tau }}}_{{\mathcal {P}}}$ (Eqs. 17 to 18).

Furthermore, since $\hat{{\varvec{\tau }}}_{{\mathcal {P}}}$ is a point estimate of zero variance, the variance of the discrepancy equals the variance of the true embedding:

20 $$\begin{aligned} \mathbb {V}[{\varvec{\tau }}_{{\mathcal {P}}} - \hat{{\varvec{\tau }}}_{{\mathcal {P}}}] = \mathbb {V}[{\varvec{\tau }}_{{\mathcal {P}}}] \end{aligned}$$

Consequently, the distribution of the true embedding ${\varvec{\tau }}_{{\mathcal {P}}}$ can be expressed as:

21 $$\begin{aligned} {\varvec{\tau }}_{{\mathcal {P}}} \sim \mathcal {N}(\hat{{\varvec{\tau }}}_{{\mathcal {P}}}, {\varvec{\Sigma }}_{{\mathcal {P}}}), \end{aligned}$$

where ${\varvec{\Sigma }}_{{\mathcal {P}}} = \mathbb {V}[{\varvec{\tau }}_{{\mathcal {P}}} - \hat{{\varvec{\tau }}}_{{\mathcal {P}}}]$ is the covariance matrix of the discrepancy.

The assumption of a Gaussian distribution for the discrepancy between estimated and true embeddings in PCA is justified by the Central Limit Theorem (CLT). In this context, the discrepancy arises as a linear combination of contributions from individual loci, with each locus contributing independently (or nearly independently) to the total variance in the embedding space. Specifically, the variance propagation formula in Eq. 15 aggregates these independent contributions through the eigenvector projections, resulting in a summation of random variables corresponding to the variance at each locus. According to the CLT, the sum (or weighted sum) of a sufficiently large number of independent random variables with finite variance converges to a Gaussian distribution, irrespective of the original distributions of the variables. Given that PCA typically operates on genotype datasets with thousands or more loci, the high dimensionality ensures that the conditions for the CLT are met, leading to a reliable approximation of the discrepancy as Gaussian distributed.

A key prerequisite for accurately estimating the distribution of the true embedding using the described approach, along with the provided estimates for $\mathbb {E}$ and $\mathbb {V}$, is that the sample of interest, ${\varvec{z}}$, originates from a data distribution similar to the one used to compute the eigenvectors and eigenvalues. If this condition is not met, alternative methods for estimating $\mathbb {V}$ may be more appropriate.

## Results

### Computation of the PC space

The principal component (PC) space was derived from commonly used modern West Eurasian populations [19] (see Additional file 1 & 2) obtained from the Allen Ancient DNA Resource (AADR) [16]. This PC space is used for all SmartPCA projections in this work. SmartPCA reported that 540,247 variant SNPs were included in the computation of the PC space, while 57,326 invariant loci were excluded. As a result, only the variant loci were retained and used consistently throughout all analyses.

### Impact of missing loci on ancient sample projections

To evaluate the effect of missing loci on the SmartPCA projections of ancient genotype individuals, a simulation experiment was performed. Fifteen high-coverage West Eurasian ancient individuals, each with at least 90 % of known genotypes, were selected from the AADR database (see Additional file 3). The selection criteria also ensured that the projections of these embeddings were distributed across the two-dimensional PC space. This distribution allowed us to investigate whether certain regions of the PC space are more sensitive to missing data than others.

For each ancient individual, loci were randomly omitted at varying rates to simulate different levels of missing data. This process was repeated 20,000 times for each rate, generating a set of downsampled variants for each ancient individual. Both the downsampled and the original high-coverage individuals were then projected onto the first two dimensions of the PC space using SmartPCA [10]. These projections enabled a comparative analysis to quantify the effect of missing loci on the embedding positions. We will refer to the projections of the high-coverage (original) samples as reference projections and the projections of the downsampled variants as subset projections.

**Fig. 2:**
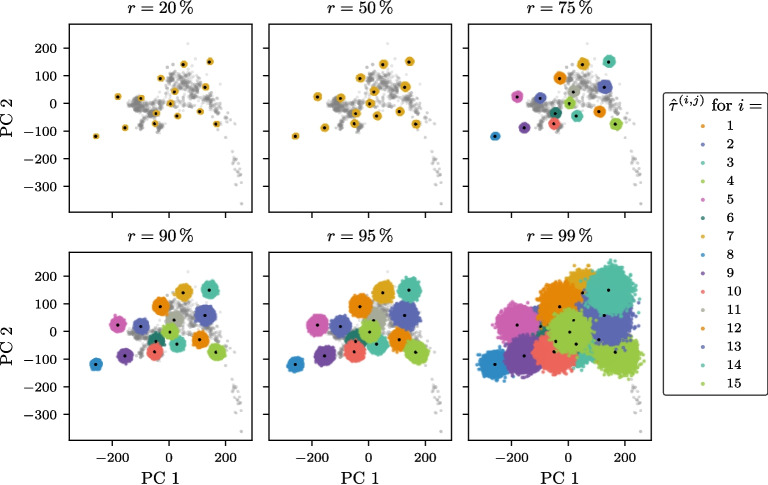
Impact of missing loci on SmartPCA projections of ancient genotype samples. The projections of the modern West Eurasian samples, used to compute the principal component (PC) subspace, are shown in gray. The black points represent the projections of 15 high-coverage ancient samples, denoted as the *true* projections $\{{\varvec{\tau }}^{(i)}\}_{i=1, \ldots , 15}$. To evaluate the impact of missing loci, loci were randomly omitted from the ancient samples at an increasing rate *r*. This process was repeated 20,000 times for each sample *i*. The resulting SmartPCA projections, $\{\hat{{\varvec{\tau }}}^{(i, j)}\}_{j=1, \ldots , 20000}$, are shown in their respective colors for each sample *i*

Figure 2 shows that as the proportion of missing genotypes increases, the spread of the subset projections around their corresponding reference projections becomes larger. Moreover, the subset projections are isotropically distributed around the reference projections, indicating that the uncertainty introduced by missing loci does not exhibit directional bias in the PC space. While slight differences in the extent of the spread are observed at different rates of missing data, the location of the reference projections within the PC space does not appear to systematically influence the degree of spread.

To quantify the impact of missing loci on SmartPCA projections, we examined the discrepancies in PC1 and PC2 for the downsampled ancient samples across varying downsampling rates (*r*). Figure S2 shows violin plots of these discrepancies, revealing a clear trend: as *r* increases, the distributions of discrepancies in both PC1 and PC2 broaden significantly, indicating that the spread of subset projections around their respective reference projections grows with the proportion of missing loci. The median of the discrepancies remains consistently at zero for all *r*, reflecting the isotropic nature of the projection spread and the absence of systematic directional bias in the PC space. However, the spread, as captured by the width of the violin plots, increases steadily with growing *r*.

### Uncertainty prediction of SmartPCA embeddings

The simulation results presented in section “Impact of missing loci on ancient sample projections” demonstrate that missing loci can lead to SmartPCA subset projections that deviate substantially from their corresponding reference projections, as illustrated in Fig. 2. The principal component (PC) scores are derived as a linear combination of eigenvectors and genotypes. Consequently, the influence of missing loci on a projection is determined by two factors: the corresponding eigenvector entries and the (unknown) genotypes at those loci.

In real-world scenarios, the genotypes at missing loci are unavailable, making it impossible to directly compute their contributions to the projection. To address this, we developed a probabilistic model that predicts a distribution for the true embedding location ${\varvec{\tau }}$, given the SmartPCA embedding $\hat{{\varvec{\tau }}}$ (for details, see section “Modeling the deviation between estimated and true embeddings in PCA”). In this model, the error analysis by Nelson *et al.* [8] is extended by incorporating assumptions on the variance structure of the PC scores. We applied variance propagation to predict distributions for true projection locations. These predictions were evaluated against empirical distributions derived from simulations with varying levels of downsampled loci, enabling a direct comparison of predicted and observed effects of missing data.

#### Uncertainty predictions for modern genotype samples

**Fig. 3:**
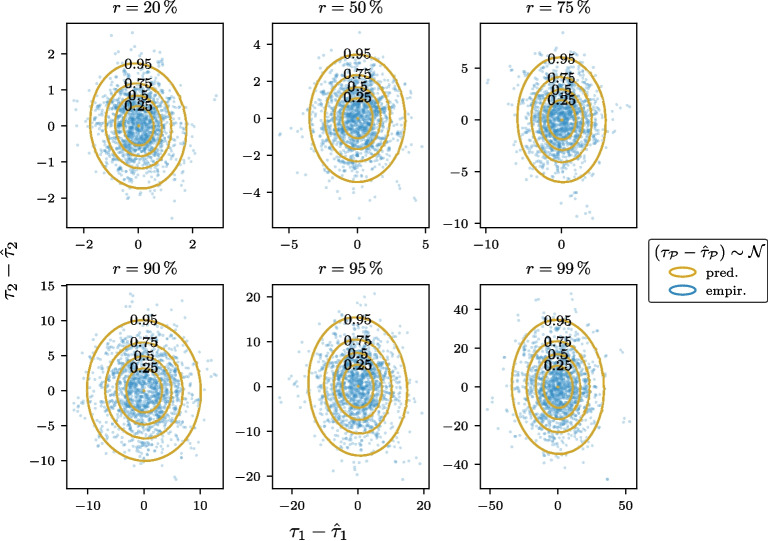
Comparison of empirically determined and predicted Gaussian discrepancy distributions for modern genotype sample embeddings. For each of 1,433 high-coverage modern West Eurasian genotype samples, a random set of loci was omitted at the specified downsampling rate *r*. The discrepancy in the first two principal components between the resulting SmartPCA sample embeddings and the reference embeddings was calculated and plotted as scatter points. From this data, the empirical Gaussian distribution was estimated and represented by blue contours at the specified quantiles. Using the corresponding sets of omitted loci, the Gaussian discrepancy distribution was predicted based on Eqs. 11-15, with the predicted contours shown in gold overlaying the blue contours

In this experiment, we simulated missing data by selecting a single set of loci to be omitted for each downsampling rate *r*. These loci were removed from all 1,433 full-coverage modern West Eurasian individuals, which were subsequently projected using SmartPCA. The differences between the resulting embedding estimates, $\hat{{\varvec{\tau }}}$, and the reference embeddings, ${\varvec{\tau }}$, were computed for the first two principal components and plotted as scatter points (Fig. 3, blue points). From this distribution of discrepancies, we calculated the mean and covariance, which were used to define an empirical Gaussian distribution.

To predict the distribution of the discrepancy between the true and estimated projections, we leveraged the fact that the same 1433 modern West Eurasian individuals were used to compute the PC axes. This ensures that the eigenvalues of the axes provide valid estimates for the variances of the PC scores. Furthermore, assuming that the mean of the PC scores is zero aligns with the PCA framework. These moments were propagated through the discrepancy function (Eqs. 11-15) to derive a predicted Gaussian distribution for the discrepancy.

As illustrated in Fig. 3, the empirical distributions, visualized as blue covariance ellipses centered on zero, show strong agreement with the predicted distributions (depicted in gold) across all downsampling rates *r*. Notably, as the proportion of missing loci increases, the empirical spread of the subset projections widens, consistent with the predictions derived from the variance propagation method.

To quantify the similarity between the empirically observed and predicted distributions, we computed the Kullback-Leibler (KL) divergence between the two distributions obtained from modern genotype samples for multiple sets of omitted loci at varying downsampling rates. The results demonstrate that the mean KL divergence remains close to zero across all rates of missing data, indicating a strong concordance between the empirical and predicted distributions (Figure S3).

The results obtained serve as a proof of concept, as the mean and variance structure of the PC scores for the modern individuals used in this experiment were known. This is because the modern samples under investigation originated from the same distribution that was used to compute the PCA itself. In the next section, we extend this approach to predict the discrepancy distribution for out-of-distribution samples, such as ancient genotype samples.

#### Uncertainty predictions for ancient genotype samples

**Fig. 4:**
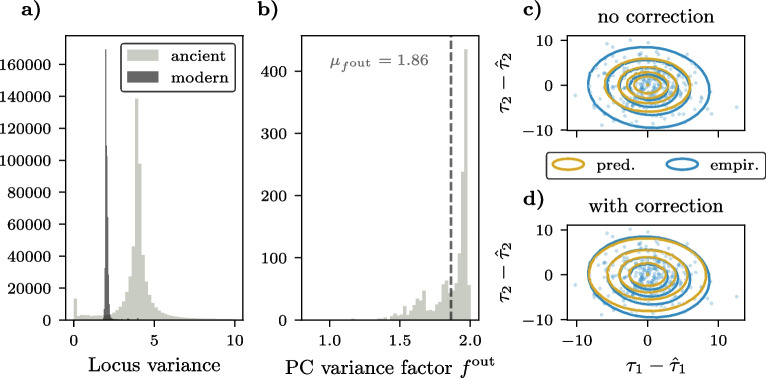
Locus variances for ancient and modern samples and their influence on prediction accuracy. **a** Distribution of 540,247 locus variances across 1,433 modern and 6,627 ancient samples. **b** Distribution of principal component (PC) variance factors, quantifying the relative variance of PC scores in ancient embeddings compared to modern ones. **c**,**d** Comparison of empirically determined and predicted Gaussian discrepancy distributions for ancient genotype sample embeddings. For 304 high-coverage ancient West Eurasian genotype samples, a random set of loci was omitted at the specified downsampling rate *r*, with results shown here for $r=0.75$. Discrepancies in the first two principal components between the resulting SmartPCA sample embeddings and the reference embeddings were calculated and plotted as scatter points. Empirical Gaussian distributions were estimated from these discrepancies and visualized as blue contours at the specified quantiles. **c** Gaussian discrepancy distributions were predicted using the corresponding set of omitted loci and the model described in Eqs. 11-15. **d** Variance adjustments (Eq. 24) were incorporated into the prediction framework to refine the predictions

Across the examined 1,433 West Eurasian modern and 6,627 ancient samples, we observed higher genotype variation at most loci in ancient samples compared to modern ones (Fig. 4a). This difference arises from the pseudohaploid SNP calling approach for ancient data, which randomly samples one allele at each locus due to the low coverage of degraded genomic material. The binary state (homozygous reference or homozygous alternative) naturally increases the variance compared to the EIGENSTRAT encoding of modern genotype samples [22].

The predictive framework developed for modern samples provides a baseline for modeling PCA embedding uncertainty. However, ancient samples introduce additional challenges due to their higher genotype variance and distinct data characteristics. To address these challenges, we extend the variance propagation approach by incorporating adjustments specific to ancient samples. In the following, we outline these adjustments and evaluate their impact on predicting the discrepancy distribution.

To this end, we selected 304 high-coverage out of the 6,627 ancient samples and simulated missing data by omitting a single set of loci for each downsampling rate *r*. As shown in Fig. 4c (for a downsampling rate of 0.75), the discrepancies between the SmartPCA embeddings and the reference embeddings are significantly larger for ancient samples compared to modern ones (see upper right subplot in Fig. 3 for comparison). When using the same assumptions about the PC score means and variances as those applied to modern samples to predict the discrepancy distribution, the variance of the predicted distribution is notably underestimated (Fig. 4c, golden contours). These findings underscore the importance of incorporating the increased genotype variation observed in ancient samples to accurately model their discrepancy distributions.

To account for the increased genotype variation observed in ancient samples, we introduced a factor $f^{\text {in}}_i$ for each locus *i*, which quantifies the relative variance of genotypes in ancient versus modern samples. Specifically, $f^{\text {in}}_i$ is defined as:

22 $$\begin{aligned} f^{\text {in}}_i = \frac{\mathbb {V}_{\text {ancient}}[i]}{\mathbb {V}_{\text {modern}}[i]} \end{aligned}$$

where $\mathbb {V}_{\text {ancient}}[i]$ and $\mathbb {V}_{\text {modern}}[i]$ denote the variances of genotypes at locus *i*, estimated across all 6,627 ancient and 1,433 modern West Eurasian samples, respectively.

To translate this locus-specific variance scaling into the PCA space, we projected the vector of factors ${\varvec{f}}^{\text {in}} \in \mathbb {R}^{D}$ (where *D* is the total number of loci) onto the PC axes using the eigenvector matrix ${\varvec{V}}$. The resulting projected factors ${\varvec{f}}^\text {out}$ estimate the relative variance of PC scores in ancient embeddings relative to modern ones:

23 $$\begin{aligned} {\varvec{f}}^\text {out} = \text {diag}({\varvec{V}}^\intercal {\varvec{f}}^\text {in} {\varvec{V}}) \end{aligned}$$

Here, $\text {diag}(\cdot )$ represents the operation of forming a diagonal matrix from a vector. The resulting ${\varvec{f}}^\text {out} \in \mathbb {R}^Q$ provides the variance adjustments required to account for ancient samples’ increased genotype variation when predicting their discrepancy distributions.

The distribution of these variance factors (Fig. 4b) reveals that ancient embeddings exhibit up to a two-fold increase in variance for their PC scores and a mean of 1.86. Incorporating this variance adjustment into the prediction framework,

24 $$\begin{aligned} \mathbb {V}[{\varvec{\tau }}_{\mathcal {Q}}] = {\varvec{f}}^\text {out} {\varvec{\Lambda }}_{\mathcal {Q}}, \end{aligned}$$

yields predicted contours that align closely with the empirical discrepancy distributions (Fig. 4d). This approach markedly improves prediction accuracy compared to using unadjusted variance assumptions, as illustrated by the misalignment of contours without correction (Fig. 4c). Furthermore, the improvement is consistent across various downsampling rates, as detailed in Figures S4 and S5. The effectiveness of the variance correction is quantitatively supported by a substantial reduction in the Kullback-Leibler (KL) divergence between the empirically derived and predicted distributions, as shown in Figure S3. These findings highlight the critical role of variance adjustments in accurately modeling PCA projection uncertainties for ancient genotype samples.

### Real-world data analysis: uncertainty predictions for ancient samples

To evaluate the performance of the proposed framework on real-world data, eight ancient individuals from the Mesolithic until early Middle Ages from the AADR database were analyzed. These individuals, spanning a range of SNP coverage levels, were specifically selected because they project to diverse locations within the West Eurasian PCA map (Table 1). The principal component (PC) space was constructed from modern West Eurasian populations, and the selected ancient samples were projected onto this space using SmartPCA. For each sample, the discrepancy distributions were predicted based on the available and missing genotypes. These predictions, centered on the reference projections, were visualized as uncertainty regions in the PCA plot.

**Table 1:** Properties of ancient test samples from the AADR

Nr.	Master ID	Locality	Period	SNP Coverage [in %]	Reference
1	STR266b	Germany	Middle Ages	4.0	[23]
2	I2014	Germany	Neolithic	1.7	[24]
3	VK470	Russia	Middle Ages	2.1	[25]
4	cth842	Turkey	Neolithic	2.8	[26]
5	I7207	Czechia	Bronze Age	70.2	[27]
6	I4884	Czechia	Bronze Age	76.3	[28]
7	I6531	Poland	Bronze Age	70.8	[28]
8	I11710	Slovakia	Iron Age	77.9	[29]

SNP coverage refers to the proportion of observed single nucleotide polymorphisms (SNPs) in the sample relative to the total of 540,247 SNPs considered in this work

The results reveal a clear distinction between samples with high SNP coverage and those with low SNP coverage. Four of the samples, characterized by high coverage (less than 30 % missingness), exhibit narrow discrepancy distributions, indicating high confidence in their embeddings. In contrast, the other four samples, which have substantially lower SNP coverage (at least 96 % missingness), display broader discrepancy distributions. This increased uncertainty reflects the reduced reliability of projections for sparse samples.

**Fig. 5:**
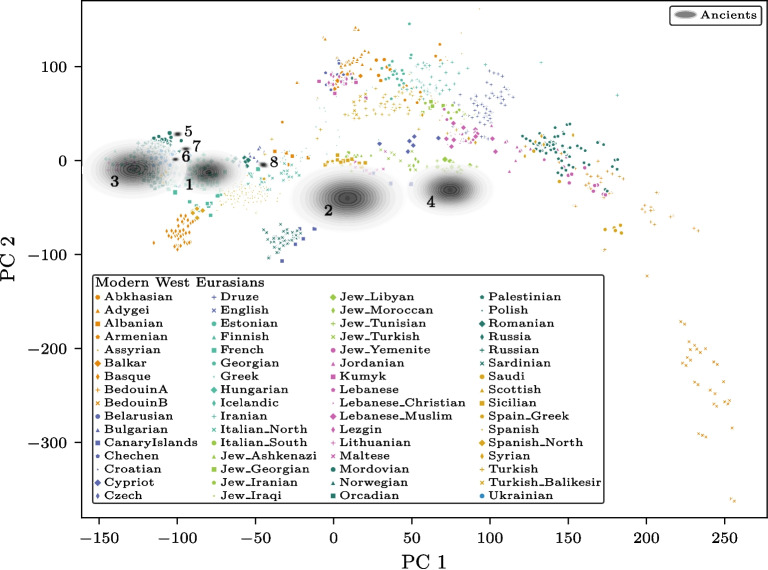
West Eurasian PCA map showing modern individuals, color-coded by their locality, projected onto the first two principal components. Predicted embedding distributions (see section “Modeling the deviation between estimated and true embeddings in PCA”) are displayed in gray for eight ancient samples with varying SNP coverage (detailed in Table 1). These distributions illustrate the uncertainty in the projections, with narrower distributions corresponding to higher SNP coverage and broader distributions indicating lower coverage. The figure was generated using TrustPCA (see section “TrustPCA: a user-friendly tool to assess SmartPCA projection uncertainties”)

The PCA plot in Fig. 5 overlays the predicted discrepancy distributions onto the modern sample projections. The narrow distributions of high-coverage samples appear tightly clustered around their reference projections, whereas the low-coverage samples show a visibly expanded spread. Notably, these broader distributions intersect with multiple regions of the PC space, highlighting the potential ambiguity in interpreting the ancestry of sparse samples. This analysis underscores the importance of accounting for embedding uncertainty when interpreting PCA projections of ancient genotype samples with missing data.

### TrustPCA: a user-friendly tool to assess SmartPCA projection uncertainties

As ancient genomics advances and PCA remains central in human population genetics, addressing uncertainties in SmartPCA projections caused by missing data is crucial. To this end, TrustPCA, a Tool for Reliability and Uncertainty in SmartPCA, was developed to apply the prediction framework from section “Modeling the deviation between estimated and true embeddings in PCA” in a user-friendly way.

Implemented in Python (v3.9) with the Streamlit framework [30], TrustPCA features an intuitive web interface. Users can upload genotype data in EIGENSTRAT format or explore preloaded datasets. Summary statistics, such as the number of missing loci per sample, are calculated and presented.

Using the PC space derived from modern West Eurasian genotype data (see section “Computation of the PC space”), TrustPCA enables users to project ancient samples similarly to SmartPCA. It predicts projection uncertainties based on missing loci in the provided samples, visualizing these as uncertainty ellipses on PC plots, representing different confidence levels for sample placements.

All outputs, including statistical summaries and visualizations, can be exported as PDFs for easy integration into research workflows. TrustPCA is openly available at https://trustpca-tuevis.cs.uni-tuebingen.de/, with the source code accessible at https://github.com/Integrative-Transcriptomics/TrustPCA/tree/main.

## Discussion

In this study, we systematically examined the effects of missing data on the reliability of SmartPCA projections for ancient human genotype samples and developed a probabilistic framework to quantify the resulting embedding uncertainty. Through simulations, we demonstrated that as the proportion of missing SNPs increases, the accuracy of SmartPCA projections decreases (see Fig. 2). This spread becomes more pronounced in cases of extreme SNP sparsity (see Figure S2). As illustrated in Figure S1, a substantial number of West Eurasian ancient individuals in the AADR dataset exhibit low SNP coverage. Specifically, approximately 15.2 % of the ancient individuals have a SNP coverage below $10\,\%$, making them especially susceptible to inaccurate SmartPCA projections.

When repeating the simulation with out-of-distribution archaic human individuals—such as Neanderthals or Denisovans—the spread of subset projections is significantly larger compared to typical ancient samples (Figure S6). This result highlights a higher degree of uncertainty in embedding positions for individuals with little genetic overlap with the modern reference population.

By analyzing real-world ancient samples from the AADR database [16], we have shown that our probabilistic model provides reliable estimates of the discrepancy distributions, offering a quantitative measure of the uncertainty in PCA projections (see Fig. 5). Importantly, the predictions highlight that high-SNP-coverage samples exhibit tight and reliable embedding distributions, while low-coverage samples display broader and less precise embeddings. These results underscore the limitations of interpreting PCA projections of sparse ancient samples without accounting for the inherent uncertainty introduced by missing data.

As PCA is frequently used in ancient DNA population genetics to generate hypotheses about population shifts or continuity for further testing with quantitative methods like *f*-statistics, inaccuracies in the assumptions derived from PCA placements can significantly impact subsequent analyses (e.g., [31, 32]). Specifically, misinterpretation of PCA results may lead to certain hypotheses being overlooked or inadequately tested.

The framework we present not only highlights these limitations but also offers a practical solution: with TrustPCA, a tool that integrates this probabilistic model, we provide researchers with uncertainty estimates alongside PCA projections. This approach enables more robust interpretations of PCA results and helps mitigate the risk of misinterpretations in studies of ancient population structure and ancestry.

The concerns raised by Elhaik [11] regarding the reliability of PCA in population genetics highlight the broader challenges in using this method. These include misleading projections, dependence on reference populations, and possibly incorrect interpretations. Our work addresses some of these challenges by quantifying and visualizing the uncertainty in PCA projections. By explicitly modeling the variability introduced by missing data, our probabilistic framework reduces the risk of overinterpretation and enables more robust conclusions. However, it does not resolve all previously mentioned issues. Notably, the choice of reference populations remains a critical factor influencing PCA results, and the interpretation of projections can still be subjective. Future research could investigate how reference population biases interact with data sparsity to influence PCA outcomes.

It is important to note that this study focused exclusively on the West Eurasian PCA map, which forms the basis of the majority of current human population genetic studies. While this choice allowed us to focus on a well-characterized dataset, the conclusions drawn here may not generalize to other geographic regions or datasets with distinct profiles of genetic structure. Future research should explore whether observed uncertainty patterns persist in other genetic landscapes and reference populations, particularly in regions with greater levels of genetic diversity or admixture.

A key prerequisite for accurately estimating the distribution of the true embedding using our probabilistic approach is that the sample of interest originates from a data distribution similar to the one used to compute the eigenvectors and eigenvalues. If this condition is not met, the variance of the predicted embedding distribution may be underestimated (Figure S4) or overestimated (Figure S6). For ancient individuals with a genotype variance structure different from that of the modern population, a correction for this variance disparity was applied, resulting in well-aligned predicted distributions (Figure S3 & S5).

The issue of missing data has also led to the widespread use of genotype imputation to fill in gaps in ancient datasets [33–35]. A recent study by Allentoft et al. [36] demonstrated that using fully imputed ancient genomes to construct the PCA—rather than relying solely on modern reference populations—can improve the resolution of ancient individuals in PCA space. However, while imputation can significantly improve the number of successfully genotyped SNPs, it introduces its own challenges. Imputed genotypes are probabilistic estimates based on linkage disequilibrium patterns in reference panels, and their accuracy depends heavily on the quality and compatibility of the reference panel with the sample being studied [37]. Errors in imputation, particularly for SNPs with low read coverage or in regions not well represented in the reference panel, can lead to biased PCA projections [38]. Future work could integrate imputation uncertainty into PCA models, enabling researchers to differentiate between uncertainty arising from imputed versus directly observed SNPs.

Another source of uncertainty arises from genotype calling, particularly in ancient DNA studies where low read coverage and contamination can result in ambiguous base calls [39]. Furthermore, the level of accuracy with which ancient individuals—called using pseudo-haploid genotypes—can be projected onto a map constructed from modern individuals with diploid genotype calls remains unclear. These uncertainties are compounded by the encoding of genotypes in population genetics analyses, which often assume fixed values (e.g., 0, 1, or 2 for diploid genotypes). This deterministic encoding overlooks the probabilistic nature of genotype likelihoods derived from sequencing data [40–42], introducing hidden uncertainties into downstream PCA projections. Incorporating genotype likelihoods directly into PCA models or using probabilistic representations of genotypes could enhance the robustness of PCA projections, particularly for low-SNP-coverage ancient samples.

## Conclusions

In conclusion, this work emphasizes the importance of considering projection uncertainty when applying PCA to sparse ancient genotype data. By quantifying this uncertainty, we offer researchers a powerful tool for making more reliable and reproducible inferences. However, addressing the broader challenges of PCA in population genetics, including reference population dependency and imputation bias, will require continued methodological innovation and critical evaluation of current practices.

## Supplementary Information

Supplementary Material l.

Supplementary Material 2.

Supplementary Material 3.

Supplementary Material 4.

Supplementary Material 5.

## Data Availability

The dataset analyzed during the current study is openly available from the Allen Ancient DNA Resource (AADR), version v54.1.p1: https://doi.org/10.7910/DVN/FFIDCW. Project name: TrustPCA Project home page: https://github.com/Integrative-Transcriptomics/trustpca Operating system(s): Platform independent Programming language: Python Other requirements: N/A License: GNU General Public License v3.0 Any restrictions to use by non-academics: License needed.

## References

[1] Cavalli-Sforza LL, Piazza A. Analysis of evolution: evolutionary rates, independence and treeness. Theor Popul Biol. 1975;8(2):127–65. 10.1016/0040-5809(75)90029-5. 1198349 10.1016/0040-5809(75)90029-5

[2] Hotelling H. Analysis of a complex of statistical variables into principal components. J Educ Psychol. 1933;24(417–441):498–520. 10.1037/h0071325.

[3] Pearson KLIII. On lines and planes of closest fit to systems of points in space. London Edinb Dublin Philos Mag J Sci. 1901;2(11):559–72. 10.1080/14786440109462720.

[4] McVean G. A genealogical interpretation of Principal Components Analysis. PLoS Genet. 2009;5:e1000686. 10.1371/journal.pgen.1000686. 19834557 10.1371/journal.pgen.1000686 PMC2757795

[5] Peter BM. A geometric relationship of , and -statistics with principal component analysis. Phil Trans R Soc B. 1852;2022(377):20200413. 10.1098/rstb.2020.0413. 10.1098/rstb.2020.0413 PMC9014194 35430884

[6] Hofreiter M, Serre D, Poinar HN, Kuch M, Pääbo S. Ancient DNA. Nat Rev Genet. 2001;2(5):353–9. 10.1038/35072071. 11331901 10.1038/35072071

[7] van Ginkel JR. In: Handling Missing Data in Principal Component Analysis Using Multiple Imputation. Cham: Springer International Publishing; 2023. pp. 141–61. 10.1007/978-3-031-10370-4_8.

[8] Nelson PRC, Taylor PA, MacGregor JF. Missing data methods in PCA and PLS: Score calculations with incomplete observations. Chemometr Intell Lab Syst. 1996;35:45–65. 10.1016/S0169-7439(96)00007-X.

[9] Patterson N, Price AL, Reich D. Population structure and eigenanalysis. PLoS Genet. 2006;2(12):e190. 10.1371/journal.pgen.0020190. 17194218 10.1371/journal.pgen.0020190 PMC1713260

[10] Price AL, Patterson NJ, Plenge RM, Weinblatt ME, Shadick NA, Reich D. Principal components analysis corrects for stratification in genome-wide association studies. Nat Genet. 2006;38(8):904–9. 10.1038/ng1847. 16862161 10.1038/ng1847

[11] Elhaik E. Principal Component Analyses (PCA)-based findings in population genetic studies are highly biased and must be reevaluated. Sci Rep. 2022;12:14683. 10.1038/s41598-022-14395-4. 36038559 10.1038/s41598-022-14395-4 PMC9424212

[12] van Waaij J, Li S, Garcia-Erill G, Albrechtsen A, Wiuf C. Evaluation of population structure inferred by principal component analysis or the admixture model. Genetics. 2023;225(2):iyad157. 10.1093/genetics/iyad157. 37611212 10.1093/genetics/iyad157

[13] Tian C, Plenge RM, Ransom M, Lee A, Villoslada P, Selmi C, et al. Analysis and application of European genetic substructure using 300 K SNP information. PLoS Genet. 2008;4(1):e4. 10.1371/journal.pgen.0040004. 18208329 10.1371/journal.pgen.0040004 PMC2211544

[14] Tian C, Kosoy R, Nassir R, Lee A, Villoslada P, Klareskog L, et al. European population genetic substructure: further definition of ancestry informative markers for distinguishing among diverse European ethnic groups. Mol Med. 2009;15:371–83. 10.2119/molmed.2009.00094. 19707526 10.2119/molmed.2009.00094 PMC2730349

[15] Patterson N, Moorjani P, Luo Y, Mallick S, Rohland N, Zhan Y, et al. Ancient admixture in human history. Genetics. 2012;192(3):1065–93. 10.1534/genetics.112.145037. 22960212 10.1534/genetics.112.145037 PMC3522152

[16] Mallick S, Reich D. The Allen Ancient DNA Resource (AADR): A curated compendium of ancient human genomes. Harvard Dataverse; 2023. 10.7910/DVN/FFIDCW. 10.1038/s41597-024-03031-7 PMC10858950 38341426

[17] Görtler J, Spinner T, Streeb D, Weiskopf D, Deussen O. Uncertainty-aware principal component analysis. IEEE Trans Vis Comput Graph. 2019;26(1):822–31. 10.1109/TVCG.2019.2934812. 31603820 10.1109/TVCG.2019.2934812

[18] Zabel S, Hennig P, Nieselt K. VIPurPCA: Visualizing and propagating uncertainty in principal component analysis. IEEE Trans Vis Comput Graph. 2023;30(4):2011–22. 10.1109/TVCG.2023.3345532. 10.1109/TVCG.2023.3345532 38127602

[19] Lazaridis I, Patterson N, Mittnik A, et al. Ancient human genomes suggest three ancestral populations for present-day Europeans. Nature. 2014;513(7518):409–13. 10.1038/nature13673. 25230663 10.1038/nature13673 PMC4170574

[20] Purcell S, Neale B, Todd-Brown K, Thomas L, Ferreira MAR, Bender D, et al. PLINK: a toolset for whole-genome association and population-based linkage analyses. Am J Hum Genet. 2007;81:559–75. 10.1086/519795. 17701901 10.1086/519795 PMC1950838

[21] Taylor JR. An Introduction to Error Analysis: The Study of Uncertainties in Physical Measurements. University Science Books, P.O. Box 605 Herndon, VA US; 1997.

[22] Barlow A, Hartmann S, Gonzalez J, Hofreiter M, Paijmans JLA. Consensify: A Method for Generating Pseudohaploid Genome Sequences from Palaeogenomic Datasets with Reduced Error Rates. Genes (Basel). 2020;11(1):50. 10.3390/genes11010050. 31906474 10.3390/genes11010050 PMC7017230

[23] Veeramah KR, Rott A, Groß M, van Dorp L, López S, Kirsanow K, et al. Population genomic analysis of elongated skulls reveals extensive female-biased immigration in Early Medieval Bavaria. Proc Natl Acad Sci. 2018;115(13):3494–9. 10.1073/pnas.1719880115. 29531040 10.1073/pnas.1719880115 PMC5879695

[24] Lipson M, Szécsényi-Nagy A, Mallick S, Pósa A, Stégmár B, Keerl V, et al. Parallel palaeogenomic transects reveal complex genetic history of early European farmers. Nature. 2017;551(7680):368–72. 10.1038/nature24476. 29144465 10.1038/nature24476 PMC5973800

[25] Margaryan A, Lawson DJ, Sikora M, Racimo F, Rasmussen S, Moltke I, et al. Population genomics of the Viking world. Nature. 2020;585(7825):390–6. 10.1038/s41586-021-03328-2. 32939067 10.1038/s41586-020-2688-8

[26] Yaka R, Mapelli I, Kaptan D, Doğu A, Chyleński M, Erdal ÖD, et al. Variable kinship patterns in Neolithic Anatolia revealed by ancient genomes. Curr Biol. 2021;31(11):2455–68. 10.1016/j.cub.2021.03.050. 33857427 10.1016/j.cub.2021.03.050 PMC8210650

[27] Narasimhan VM, Patterson N, Moorjani P, Rohland N, Bernardos R, Mallick S, et al. The formation of human populations in South and Central Asia. Science. 2019;365(6457):eaat7487. 10.1126/science.aat7487. 31488661 10.1126/science.aat7487 PMC6822619

[28] Olalde I, Brace S, Allentoft ME, Armit I, Kristiansen K, Booth T, et al. The Beaker phenomenon and the genomic transformation of northwest Europe. Nature. 2018;555(7695):190–6. 10.1038/nature25738. 29466337 10.1038/nature25738 PMC5973796

[29] Patterson N, Isakov M, Booth T, Büster L, Fischer CE, Olalde I, et al. Large-scale migration into Britain during the Middle to Late Bronze Age. Nature. 2022;601(7894):588–94. 10.1038/s41586-021-04287-4. 34937049 10.1038/s41586-021-04287-4 PMC8889665

[30] Streamlit. Streamlit: Open-source app framework for Machine Learning and Data Science. 2024. https://streamlit.io/. Accessed 20 May 2025.

[31] Wang CC, Reinhold S, Kalmykov A, Wissgott A, Brandt G, Jeong C, et al. Ancient human genome-wide data from a 3000-year interval in the Caucasus corresponds with eco-geographic regions. Nat Commun. 2019;10(1):590. 10.1038/s41467-018-08220-8. 30713341 10.1038/s41467-018-08220-8 PMC6360191

[32] Ghalichi A, Reinhold S, Rohrlach AB, Kalmykov AA, Childebayeva A, Yu H, et al. The rise and transformation of Bronze Age pastoralists in the Caucasus. Nature. 2024;1–9. 10.1038/s41586-024-08113-5. 10.1038/s41586-024-08113-5 PMC11602729 39478221

[33] Browning BL, Zhou Y, Browning SR. A one-penny imputed genome from next-generation reference panels. Am J Hum Genet. 2018;103(3):338–48. 10.1016/j.ajhg.2018.07.015. 30100085 10.1016/j.ajhg.2018.07.015 PMC6128308

[34] Howie BN, Donnelly P, Marchini J. A flexible and accurate genotype imputation method for the next generation of genome-wide association studies. PLoS Genet. 2009;5(6):e1000529. 10.1371/journal.pgen.1000529. 19543373 10.1371/journal.pgen.1000529 PMC2689936

[35] Rubinacci S, Ribeiro DM, Hofmeister RJ, Delaneau O. Efficient phasing and imputation of low-coverage sequencing data using large reference panels. Nat Genet. 2021;53(1):120–6. 10.1038/s41588-020-00756-0. 33414550 10.1038/s41588-020-00756-0

[36] Allentoft ME, Sikora M, Refoyo-Martínez A, Irving-Pease EK, Fischer A, Barrie W, et al. Population genomics of post-glacial western Eurasia. Nature. 2024;625(7994):301–11. 10.1038/s41586-023-06865-. 38200295 10.1038/s41586-023-06865-0 PMC10781627

[37] Ausmees K, Sanchez-Quinto F, Jakobsson M, Nettelblad C. An empirical evaluation of genotype imputation of ancient DNA. G3 Genes Genomes Genet. 2022;12(6):jkac089. 10.1093/g3journal/jkac089. 10.1093/g3journal/jkac089 PMC9157144 35482488

[38] Sousa da Mota B, Rubinacci S, Cruz Dávalos DI, G Amorim CE, Sikora M, Johannsen NN, et al. Imputation of ancient human genomes. Nat Commun. 2023;14(1):3660. 10.1038/s41467-023-39202-0. 37339987 10.1038/s41467-023-39202-0 PMC10282092

[39] Orlando L, Allaby R, Skoglund P, Sarkissian CD, Stockhammer PW, Ávila-Arcos MC, et al. Ancient DNA analysis. Nat Rev Methods Prim. 2021;1(1). 10.1038/s43586-020-00011-0.

[40] Nielsen R, Paul JS, Albrechtsen A, Song YS. Genotype and SNP calling from next-generation sequencing data. Nat Rev Genet. 2011;12(6):443–51. 10.1038/nrg2986. 21587300 10.1038/nrg2986 PMC3593722

[41] Nielsen R, Korneliussen T, Albrechtsen A, Li Y, Wang J. SNP calling, genotype calling, and sample allele frequency estimation from new-generation sequencing data. PloS ONE. 2012. 10.1371/journal.pone.0037558. 10.1371/journal.pone.0037558 PMC3404070 22911679

[42] Korneliussen TS, Albrechtsen A, Nielsen R. ANGSD: analysis of next generation sequencing data. BMC Bioinformatics. 2014;15:1–13. 10.1186/s12859-014-0356-4. 25420514 10.1186/s12859-014-0356-4 PMC4248462

