# Supplementary material for: A probabilistic approach to visualize the effect of missing data on PCA in ancient human genomics
Source: BMC Genomics. 2025 May 27;26:537. doi: 10.1186/s12864-025-11728-1 (PMC12108005; doi:10.1186/s12864-025-11728-1)
Supplement: Supplementary file 5 — Supplementary Material 5. [file 12864_2025_11728_MOESM5_ESM.pdf]

Supplemental Material for the paper "A Probabilistic Approach to Visualize the Effect of Missing Data on PCA in Ancient Human Genomics"

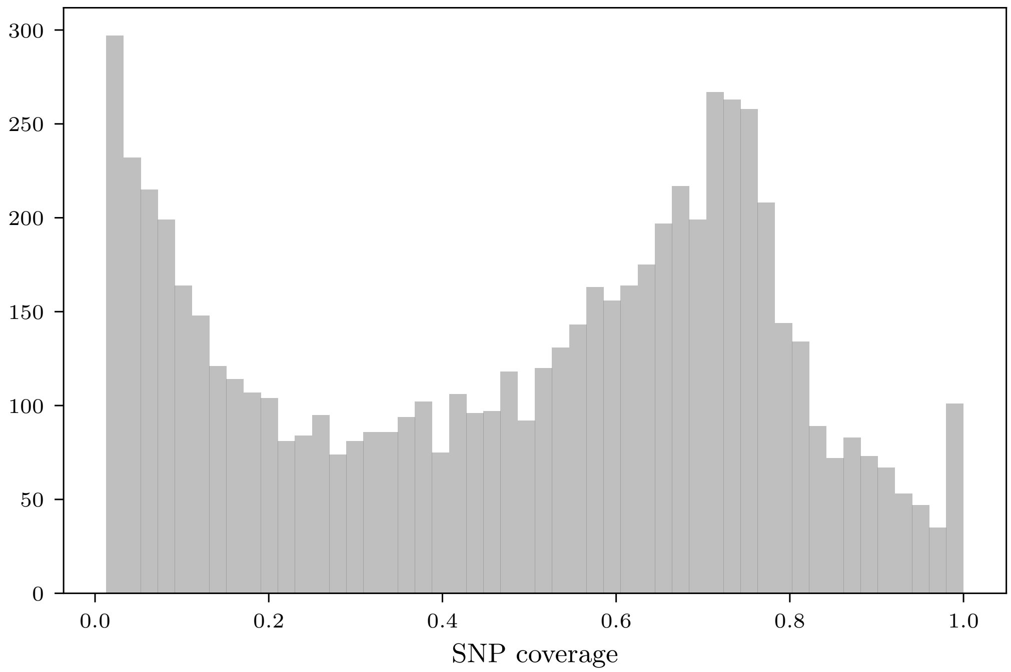

Figure S1: Histogram of SNP coverage across the 6,627 West Eurasian human individuals from the Allen Ancient DNA Resource included in this study. SNP coverage is defined as the proportion of successfully genotyped single nucleotide polymorphisms (SNPs) per individual, relative to the total of 540,247 SNPs analyzed in this work.

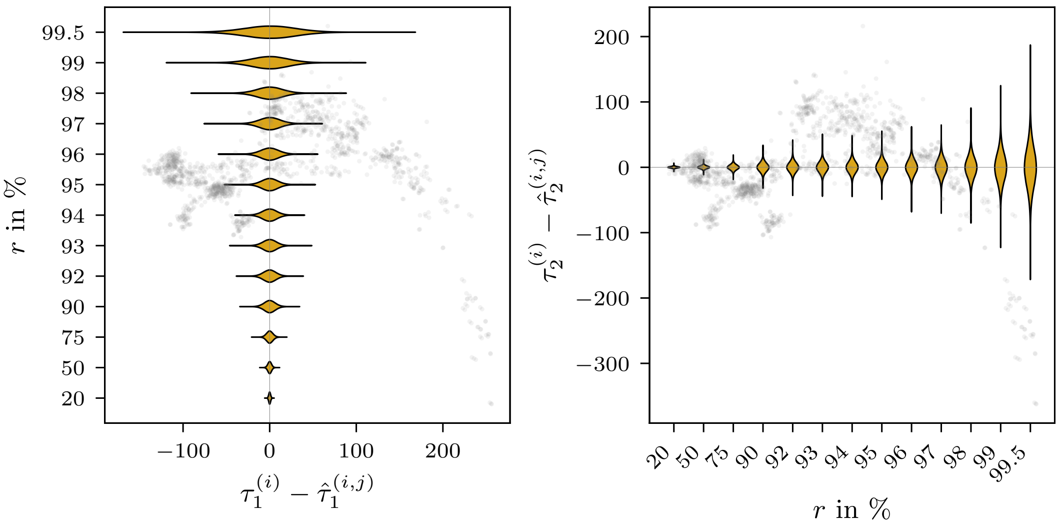

Figure S2: Violin plots of discrepancy distributions in PC1 and PC2 at varying downsampling rates (r). To assess the impact of missing loci, loci were randomly omitted from 15 high-coverage ancient samples at progressively increasing rates (r). For each sample i, this process was repeated 20,000 times. The discrepancies between the resulting SmartPCA projections $\{\hat{\boldsymbol{\tau}}^{(i,j)}\}_{j=1,\ldots,20000}$ and their respective reference projection $\boldsymbol{\tau}^{(i)}$ were computed. The component-wise distributions of these discrepancies are visualized as violin plots, illustrating how the spread of discrepancies in PC1 and PC2 changes with increasing downsampling rates. To provide a reference scale for the magnitude of $\boldsymbol{\tau}^{(i)} - \hat{\boldsymbol{\tau}}^{(i,j)}$, the map of modern PCA projections is shown in the background. Note that only the corresponding axis (left: PC1, right: PC2) is meaningful for interpreting the spread of discrepancies.

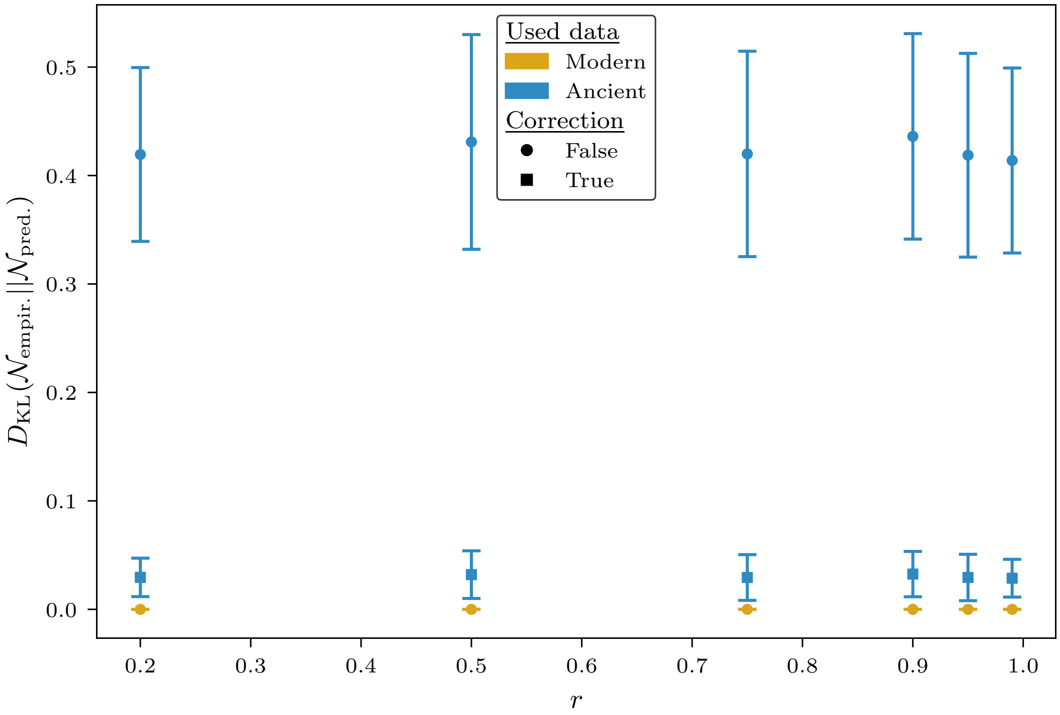

Figure S3: Kullback-Leibler (KL) divergence between empirical and predicted Gaussian discrepancy distributions. High-coverage modern and ancient samples were analyzed by randomly omitting loci at a specified rate r. For each sample, the discrepancy in the first two principal components between the resulting SmartPCA embeddings and the reference embeddings was computed. From these discrepancies, the empirical Gaussian distribution was estimated. Using the same sets of omitted loci, the Gaussian discrepancy distribution was predicted based on Equations (11)-(21). The KL divergence between the empirical and predicted distributions was then calculated. This process was repeated for 100 random draws of missing loci sets, with the mean KL divergence and standard deviation plotted to summarize the results. Marker types indicate whether a variance adjustment was applied during the prediction of the distribution.

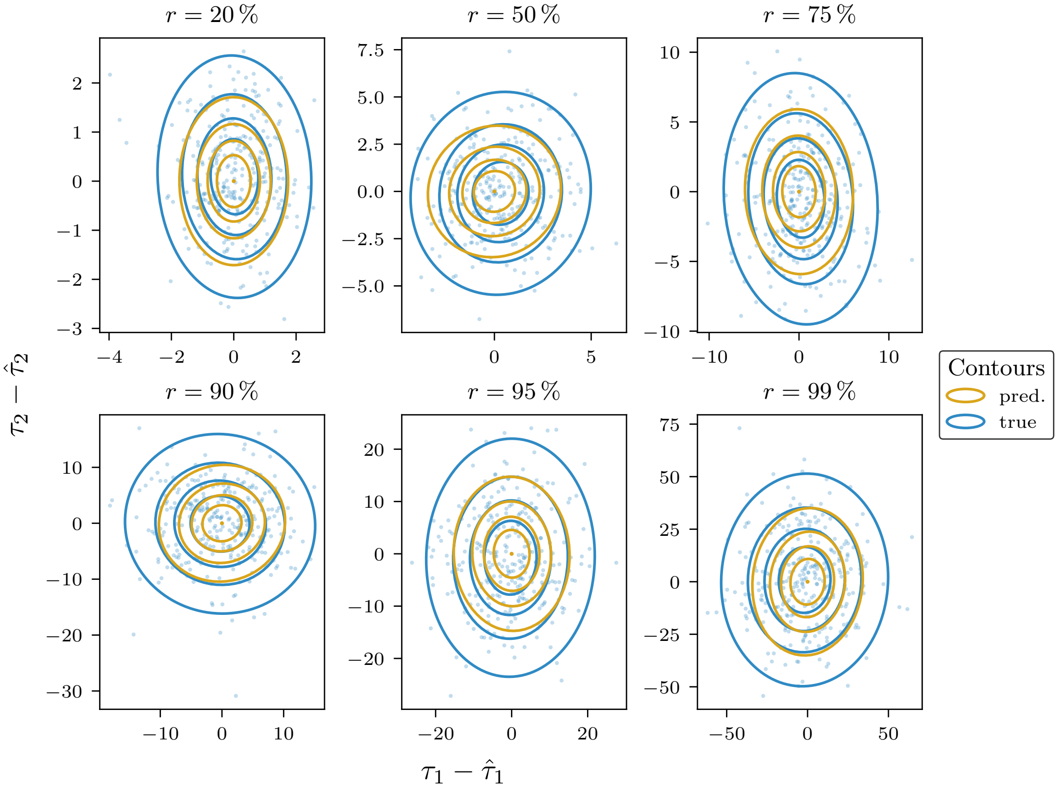

Figure S4: Comparison of empirically determined and predicted Gaussian discrepancy distributions for ancient genotype subset embeddings. For each of 304 high-coverage ancient West Eurasian genotype samples, a random set of loci was omitted at the specified downsampling rate r. The discrepancy in the first two principal components between the resulting SmartPCA subset embeddings and the reference embeddings was calculated and plotted as scatter points. From this data, the empirical Gaussian distribution was estimated and represented by blue contours at the specified quantiles. Using the corresponding sets of omitted loci, the Gaussian discrepancy distribution was predicted based on Equations (11)-(21), with the predicted contours shown in gold overlaying the blue contours. These predictions were made without adjustments for the increased variance typically observed in ancient samples compared to modern samples.

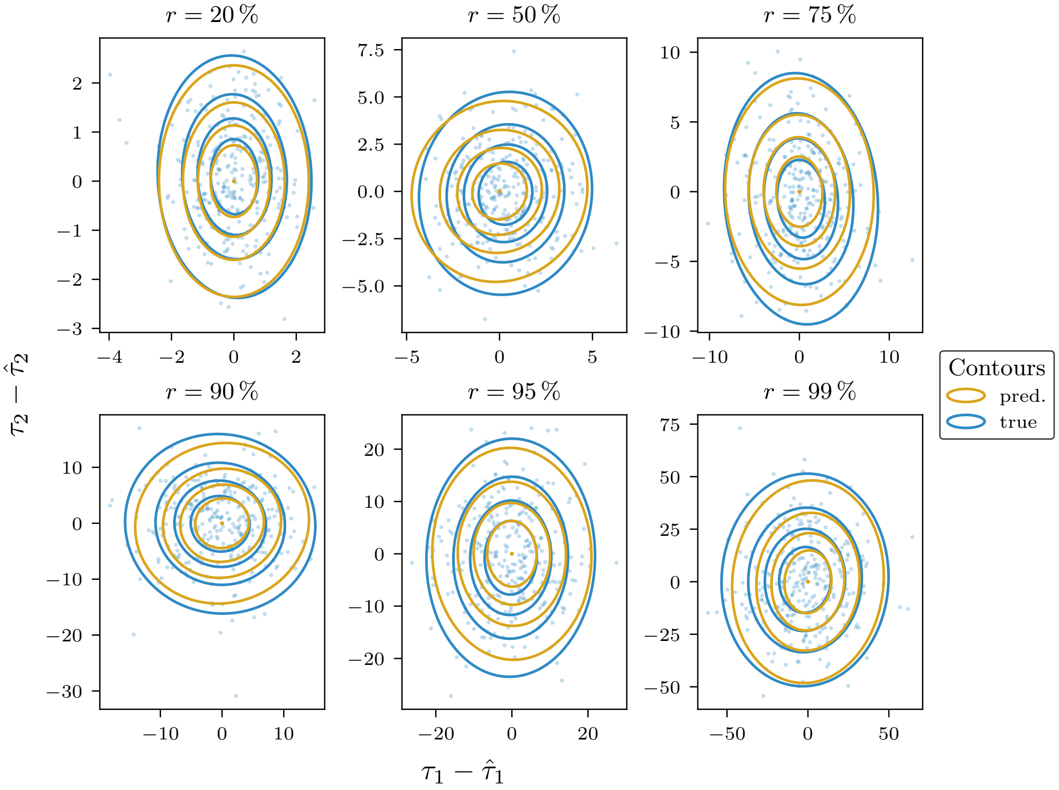

Figure S5: Comparison of empirically determined and predicted Gaussian discrepancy distributions for ancient genotype subset embeddings. For each of 304 high-coverage ancient West Eurasian genotype samples, a random set of loci was omitted at the specified downsampling rate r. The discrepancy in the first two principal components between the resulting SmartPCA subset embeddings and the reference embeddings was calculated and plotted as scatter points. From this data, the empirical Gaussian distribution was estimated and represented by blue contours at the specified quantiles. Using the corresponding sets of omitted loci, the Gaussian discrepancy distribution was predicted based on Equations (11)-(21), with the predicted contours shown in gold overlaying the blue contours. Variance adjustments were incorporated into the prediction framework to account for the increased variance typically observed in ancient samples compared to modern ones.

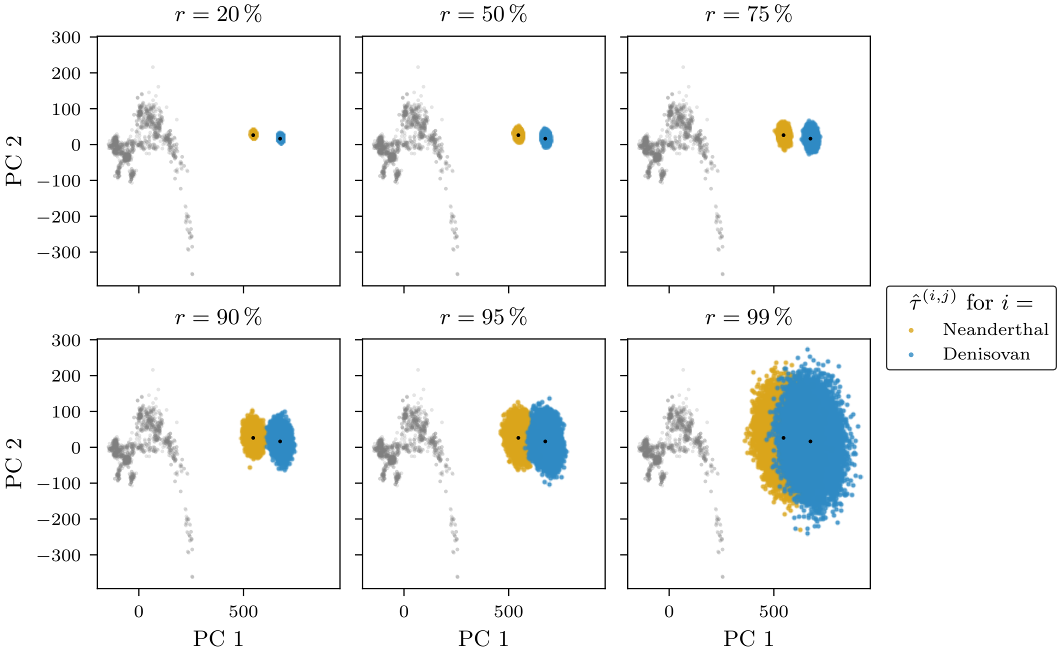

Figure S6: Impact of missing loci on SmartPCA projections of out-of-distribution ancient genotype samples. The projections of the modern West Eurasian samples, used to compute the principal component (PC) subspace, are shown in gray. The black point represents the projection of the high-coverage ancient individuals (Neanderthal & Denisovan), considered as the *true* projection $\{\boldsymbol{\tau}^{(i)}\}$. To evaluate the impact of missing loci, loci were randomly omitted from the ancient sample at an increasing rate r. This process was repeated 20,000 times. The resulting SmartPCA projections, $\{\hat{\boldsymbol{\tau}}^{(i,j)}\}_{j=1,\ldots,20000}$, are shown in orange and blue for the respective individuals.
